# Untargeted Metabolomic Profiling of the Correlation Between Prognosis Differences and PD-1 Expression in Sepsis: A Preliminary Study

**DOI:** 10.3389/fimmu.2021.594270

**Published:** 2021-04-01

**Authors:** Y. Bu, H. Wang, X. Ma, C. Han, X. Jia, J. Zhang, Y. Liu, Y. Peng, M. Yang, K. Yu, C. Wang

**Affiliations:** ^1^Department of Critical Care Medicine, Harbin Medical University Cancer Hospital, Harbin, China; ^2^Department of Critical Care Medicine, The Second Affiliated Hospital of Harbin Medical University, Harbin, China; ^3^Department of Critical Care Medicine, The First Affiliated Hospital of Harbin Medical University, Harbin, China

**Keywords:** untargeted metabolomics, sepsis, prognosis, PD-1, differential metabolites

## Abstract

**Objectives:** The mortality rate of sepsis remains very high. Metabolomic techniques are playing increasingly important roles in diagnosis and treatment in critical care medicine. The purpose of our research was to use untargeted metabolomics to identify and analyze the common differential metabolites among patients with sepsis with differences in their 7-day prognosis and blood PD-1 expression and analyze their correlations with environmental factors.

**Methods:** Plasma samples from 18 patients with sepsis were analyzed by untargeted LC-MS metabolomics. Based on the 7-day prognoses of the sepsis patients or their levels of PD-1 expression on the surface of CD4+ T cells in the blood, we divided the patients into two groups. We used a combination of multidimensional and monodimensional methods for statistical analysis. At the same time, the Spearman correlation analysis method was used to analyze the correlation between the differential metabolites and inflammatory factors.

**Results:** In the positive and negative ionization modes, 16 and 8 differential metabolites were obtained between the 7-day death and survival groups, respectively; 5 and 8 differential metabolites were obtained between the high PD-1 and low PD-1 groups, respectively. We identified three common differential metabolites from the two groups, namely, PC (P-18:0/14:0), 2-ethyl-2-hydroxybutyric acid and glyceraldehyde. Then, we analyzed the correlations between environmental factors and the common differences in metabolites. Among the identified metabolites, 2-ethyl-2-hydroxybutyric acid was positively correlated with the levels of IL-2 and lactic acid (Lac) (*P* < 0.01 and *P* < 0.05, respectively).

**Conclusions:** These three metabolites were identified as common differential metabolites between the 7-day prognosis groups and the PD-1 expression level groups of sepsis patients. They may be involved in regulating the expression of PD-1 on the surface of CD4+ T cells through the action of related environmental factors such as IL-2 or Lac, which in turn affects the 7-day prognosis of sepsis patients.

## Introduction

Sepsis is a systemic inflammatory response caused by infection, leading to organ dysfunction and death. In recent years, the mortality rate of sepsis has remained very high. Early detection of sepsis is important, and diagnosis and prognostic evaluation are still challenging. The organ dysfunction and lethality caused by sepsis are attributed to the interactions between proinflammatory and anti-inflammatory factors (1). Studies have shown that immunosuppression is the main cause of death in patients with sepsis. The occurrence of immunosuppression is mainly regulated by cellular immunity, and T lymphocyte failure and apoptosis occur. Therefore, reducing the apoptosis rate of lymphocytes and improving the function of lymphocytes can reduce mortality in patients with sepsis (2, 3). PD-1 is one of the most studied immune checkpoints in recent years. PD-1 is mainly induced on the surface of CD4+ T cells and participates in immune regulation. Studies have shown that PD-1 expression can be used to predict mortality in patients with sepsis (2, 4). Studies have shown that T cells, monocytes and neutrophils in sepsis increase the expression of PD-1 and PD-L1 and that the upregulation of PD-1 or PD-L1 expression is associated with increased mortality (2, 5–8). There are also research results showing that relatively high PD-1 and PD-L1 expression is related to mortality or a high risk of nosocomial infection (9). However, recently, in an animal experiment with cancer sepsis, compared with the sham operation group, the expression of PD-1 on the surface of CD4+ T and CD8+ T cells did not increase on day 1 and day 3 after cecal ligation and puncture (CLP). At the same time, PD-1 blocker treatment did not affect the mortality of mice with cancer sepsis (10), which was different from previous studies. There are few clinical studies on the relationship between PD-1 expression and prognosis in patients diagnosed with sepsis within 24 h after admission. Therefore, we hope to explore the relationship between the expression of PD-1 on the surface of T lymphocytes and the prognosis of patients diagnosed with sepsis within 24 h of entering the intensive care unit (ICU).

In fact, the evaluation of existing biomarkers (such as TNF-α, IL-6, and PCT) and clinical scores (such as Sequential Organ Failure Assessment (SOFA) (11) have been used in prognostic judgment, but they are not applicable in all cases (insufficient sensitivity and/or specificity) (12). The diagnosis of sepsis can also be assisted by microbial culture, but only 30 to 40% of patients with severe sepsis or septic shock produce positive test results (13). Therefore, there is still an urgent need for new and reliable methods for early diagnostic and prognostic assessments. The current understanding of the progression of sepsis involves various proinflammatory and anti-inflammatory pathways that can affect the host by activating specific pathogens (14). Metabolomic analysis can assess health and disease states by detecting and analyzing metabolites, such as those in blood, urine, and fecal samples. Metabolites can be used to discover new biomarkers for disease identification and are widely used in various diseases, including cancers (15, 16). Major diseases, such as sepsis, destroy metabolomic features. The identification of these metabolites is a useful innovation for the diagnosis and treatment of sepsis (17). Therefore, we hope to determine the differential metabolites distinguishing sepsis patients with different prognostic outcomes at 7 days and different PD-1 expression levels by untargeted metabolomics and aimed to explore the correlation between 7-day prognosis and PD-1 expression in patients with sepsis.

## Methods

### Study Design and Participants

Between October 2018 and June 2019, all consecutive adults admitted to the ICU were enrolled in this study if they had indisputable or probable sepsis in the first 24 h after ICU admission. In the study, 18 patients who met the inclusion criteria for sepsis were included. We grouped all sepsis patients according to patient prognosis and the statistical median principle. The statistical median principle was based on the statistical results for PD-1 expression levels on the surface of CD4+ T cells in flow cytometry experiments. The grouping details were as follows: a 7-day death group (7TS1, *n* = 6; median age 55.5 years), a 7-day survival group (7TS2, *n* = 12; median age 57 years), a high PD-1 expression group (PD1S1, *n* = 9; median age 59 years), and a low PD-1 expression group (PD1S2, *n* = 9; median age 52 years); the age range was 30–79 years. We collected relevant data for patients, including sex, age, and clinical laboratory examination results. All sepsis patients met the sepsis standard (SEPSIS-3) (18). The enrolled patients met the following conditions: (1) age >18 years; (2) diagnosis of sepsis in the ICU; and (3) no receipt of chemotherapy or radiation therapy. Exclusion criteria included HIV infection and neutropenia (<500 neutrophils/mm^3^). Acute Physiological and Chronic Health Assessment (APACHE II) scores and Sequential Organ Failure Assessment (SOFA) scores were determined.

### Blood Sampling

Venous blood was collected from patients with sepsis diagnosed within 24 h of ICU admission. The expression of PD-1 on the surface of CD3+, CD4+, and CD8+ T cells was detected by flow cytometry within 6 h of blood sample collection. The remaining samples were centrifuged to obtain plasma, which was stored in a freezer at −80°C and kept frozen until metabolomic analysis.

### PD-1 Detection

Samples of peripheral blood were collected in ethylenediamine tetraacetic acid (EDTA) anticoagulant tubes and transported to the laboratory at 4°C within 1 h. The erythrocytes were lysed and cells were stained by a researcher who was blinded to the clinical data. According to the manufacturer's recommendations, the following monoclonal antibodies and their isotype controls were used: phycoerythrin (PE)-labeled anti-PD1 (5 μl, Biolegend 329,906), fluorescein isothiocyanate (FITC)-labeled anti-CD3 (2 μl, Biolegend 300,306), PerCP-labeled anti-CD4 (5 μl, Biolegend 317,432), and Brilliant violet (BV) 510-labeled anti-CD8 (5 μl, Biolegend 344,732) per 100 μl of whole blood. Samples were run on the Canto Flow Cytometer (BD) and analyzed using FlowJo 10. Lymphocytes were gated by forward scatter (FSC) and side scatter (SSC), and CD4+T cells subsets were further identified by CD3+ and CD4+ staining, and CD8+ T cells were further identified by CD3+ and CD8+ staining. At least 50,000 CD3+ T lymphocytes were analyzed from each sample. The threshold was defined with the isotype control. The results are expressed as the percentage of PD-1+/CD3+ T, PD-1+/CD4+ T, and PD-1+/CD8+ T cells.

### Metabolite Extraction, Testing, and Data Processing

Fifty microliters of sample was pipetted into an EP tube, 200 μl of extraction solution (methanol: acetonitrile = 1:1 (V/V), containing isotope-labeled internal standard mixture) was added, and samples were vortexed and mixed for 30 s; Next, the tubes were ultrasonicated for 10 min (ice water bath) and allowed to stand at −40°C for 1 h. Samples were centrifuged at 4°C at 12,000 rpm for 15 min, and the supernatant was removed and placed in a sample bottle for testing. For all samples, an equal amount of supernatant was mixed into QC samples for machine testing. In this project, a Vanquish (Thermo Fisher Scientific) ultrahigh-performance liquid chromatograph was used to separate the target compounds through a Waters ACQUITY UPLC BEH Amide (2.1 mm × 100 mm, 1.7 μm) liquid chromatography column. The Phase A of the liquid chromatography is the aqueous phase, containing 25 mmol/L ammonium acetate and 25 mmol/L ammonia, and phase B is acetonitrile. The following parameters for gradient elution were used: 0~0.5 min, 95% B; 0.5~7 min, 95~65% B; 7~8 min, 65~40% B; 8~9 min, 40% B; 9~9.1 min, 40~95% B; and 9.1~12 min, 95% B. The mobile phase flow rate was: 0.5 ml/min, the column temperature was 25°C, the sample tray temperature: was 4°C, and the injection volume was 3 μl. The Thermo Q Exactive HFX mass spectrometer can collect primary and secondary mass spectrometry data with the control of the control software (Xcalibur, Thermo). The detailed parameters are as follows: sheath gas flow rate: 50 Arb; aux gas flow rate: 10 Arb; capillary temperature: 320°C; full ms resolution: 60,000; MS/MS resolution: 7,500; collision energy: 10/30/60 in NCE mode; and spray voltage: 3.5 kV (positive) or −3.2 kV (negative). After the original data were converted into mzXML format by ProteoWizard software, the self-written R program package (the kernel is XCMS) was used for peak identification, peak extraction, peak alignment and integration, and then a self-built secondary mass spectrum was used with BiotreeDB (V2.1). The database is matched for substance annotation, and the cutoff value of the algorithm score was set to 0.3. When performing peak extraction, peak alignment, and standardization of the original data, the original base peak ion (BPI), or original total ion current (TIC) chromatogram was used to initially observe the retention time reproducibility of the instrument and the measured substance quantity, and whether the chromatograms between different groups had obvious differences was determined. Then, the metabolite peak area table that can be used for analysis was obtained. To better analyze the data, we first organized the original data, which mainly includes the following steps: simulating missing value recoding in the original data (missing value recoding); the numerical simulation method used was the minimum one-half method to fill. An internal standard (IS) was used for data normalization. In addition, the instrument quality control and data control showed good results.

### Statistical Analysis

SAS9.1.3 software was used for statistical analysis. Normally distributed quantitative data are described by the mean ± standard deviation $\left(\bar{x}\pm S\right)$. Two independent-sample *t*-tests were used to compare two groups (the statistic is the *t* value). Quantitative data with a skewed distribution are described by the median and interquartile range [M (P25, P75)], and the Wilcoxon rank-sum test was used to compare two groups (the statistic is the Z value). *P* < 0.05 was considered statistically significant. Qualitative data was described by frequency (percentage). Fisher exact probability test was used for comparing the composition between the two groups (the statistic is the χ^2^ value).

This study used univariate statistics combined with multivariate statistical methods to analyze the metabolites. We used the untargeted LC-MS analysis method to obtain a data set with metabolic characteristics in positive and negative ion modes. To obtain information about the differences in metabolites between the two groups, we used a multivariate statistical analysis method, namely, orthogonal partial least squares discriminant analysis (OPLS-DA), to filter signals that were not related to the model classification, which was orthogonal signals and obtained the OPLS-DA model. The quality of the model was checked by the cross-validation method, and the validity of the model was tested by the R2Y and Q2 (representing the interpretability of the Y variable and the predictability of the model) obtained after the crossover. Then, through a permutation test, the order of categorical variable Y was randomly changed many times (1000 times) to obtain different random Q2 and R2 values, and the effectiveness of the model was further tested. The VIP value (variable importance in the projection) calculated by OPLS-DA was used to screen differential metabolites, and VIP > 1 was used for screening. At the same time, to obtain statistically significant metabolites, univariate statistical methods were also used. Depending on the normal data distribution (evaluated by the Shapiro-Wilk test), the *t*-test was used to further verify the differences in metabolites between groups. The *P*-value corrected by the Benjamini-Hochberg false discovery rate (FDR) was obtained, and the statistical significance level was set to 95% (*P* < 0.05). Spearman correlation analysis was used to analyze the correlation between environmental factors and metabolites, and *P* < 0.01 was considered highly relevant and *P* < 0.05 was considered relevant.

## Results

### Comparison of Clinical Features, Inflammatory Factors, and PD-1 Expression in Patients With Sepsis With Different Prognoses in 7 Days

**Figure 1:**
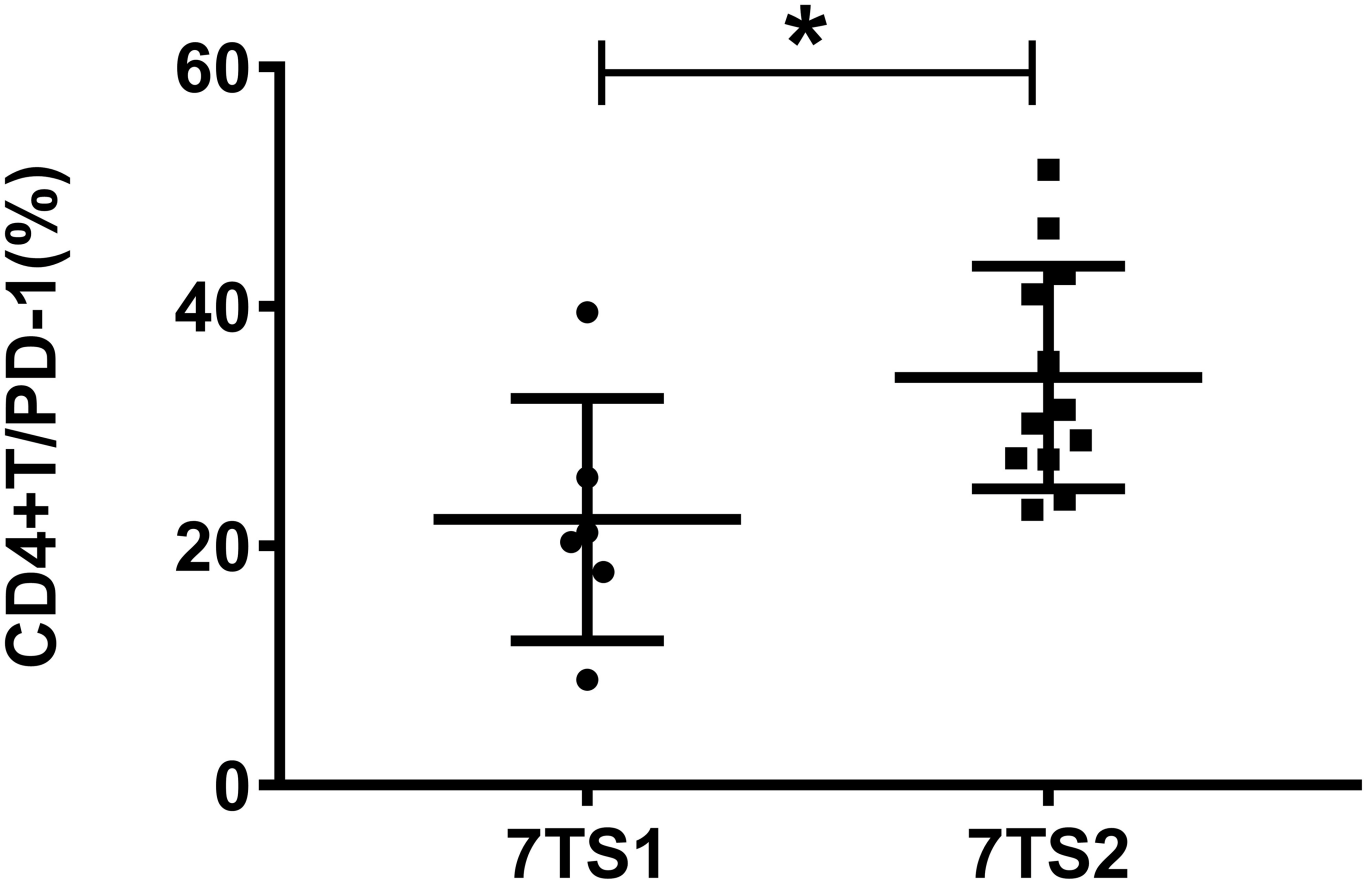
The figure shows the expression of PD-1 on CD4+ T cells between the 7-day death group (7TS1) and the survival group (7TS2). The expression of PD-1 in the 7-day death group was lower than that in the survival group (*P* < 0.05), and the difference was statistically significant. *P < 0.05.

Patients with sepsis were grouped, according to their 7-day prognosis (survival/death), and we divided them based on basic conditions including PD-1 expression, expression of six cytokines (IFN-γ, IL-1β, IL-2, IL-6, TNF-α, and IL-10), SOFA scores, APACHE II scores, procalcitonin (PCT) levels, and lactic acid (Lac) levels for comparative analysis. Statistical analysis results showed that there was a statistically significant difference in the expression of PD-1 on the surface of CD4+ T cells and the APACHE II score between the survivors and patients who died. There were no significant differences in SOFA scores or the expression levels of PCT, Lac or the six cytokines (Table 1). Compared with the survival group, the death group had a higher APACHE II score. Compared with the survival group, patients in the 7-day death group had low expression of PD-1 on the surface of CD4+ T cells (Figure 1). The flow gating strategy of PD-1 expression is shown in Supplementary Figure 1. [Supplementary Figure 1a (7TS1 group) and Supplementary Figure 1b (7TS2 group)].

**Table 1:** Comparison of relevant indexes of patients between 7-day survival group and 7-day death group.

	**Survivors (*n* = 12)**	**Non-survivors (*n* = 6)**	***t/Z/χ*^**2**^**	***P***
Gender				1.0000
Male	9 (75.00)	4 (66.67)		
Female	3 (25.00)	2 (33.33)		
Age	56.75 ± 14.03	58.33 ± 11.5	−0.24	0.8147
IFN-γ	1.86 (1.42–3.5)	2.12 (1.42–2.35)	0.0000	1.0000
IL-10	51.21 (16.41–200.95)	33.18 (11.56–63.8)	−0.7969	0.4255
IL-1β	1 (0.76–2.18)	0.88 (0.7–0.94)	−0.4257	0.6704
IL-2	1.33 (1.05–1.78)	1.48 (1.05–1.93)	0.4250	0.6708
IL-6	80.91 (19.87–3375.5)	53.41 (27.13–116.66)	−0.1405	0.8883
TNF-α	50.28 (31.08–103.66)	24.55 (15.6–157.75)	−0.4215	0.6734
CD3+T/PD-1 (%)	30.45 ± 12.63	21.62 ± 8.18	1.55	0.1416
CD4+T/PD-1 (%)	34.07 ± 9.28	22.21 ± 10.14	2.48	0.0246
CD8+T/PD-1 (%)	28.26 ± 18.81	27.34 ± 17.25	0.10	0.9213
SOFA	6.33 ± 2.87	8.00 ± 2.00	−1.27	0.2231
APACHEII	12.83 ± 3.56	17.67 ± 4.27	−2.54	0.0217
Procalcitonin (PCT)	19.80 (6.11–31.99)	4.00 (0.31–8.29)	−1.3576	0.1746
Lactic acid (Lac)	2.20 (1.05–6.20)	4.20 (2.80–8.50)	0.8902	0.3733

*The t value is a statistical variable obtained by comparing two groups with two independent sample t-tests; the Z value is a statistical variable obtained by comparing the Wilcoxon rank sum test between the two groups*.

### Metabolite Analysis and Model Validation of the 7-Day Prognosis Group and PD-1 Expression Group

**Figure 2:**
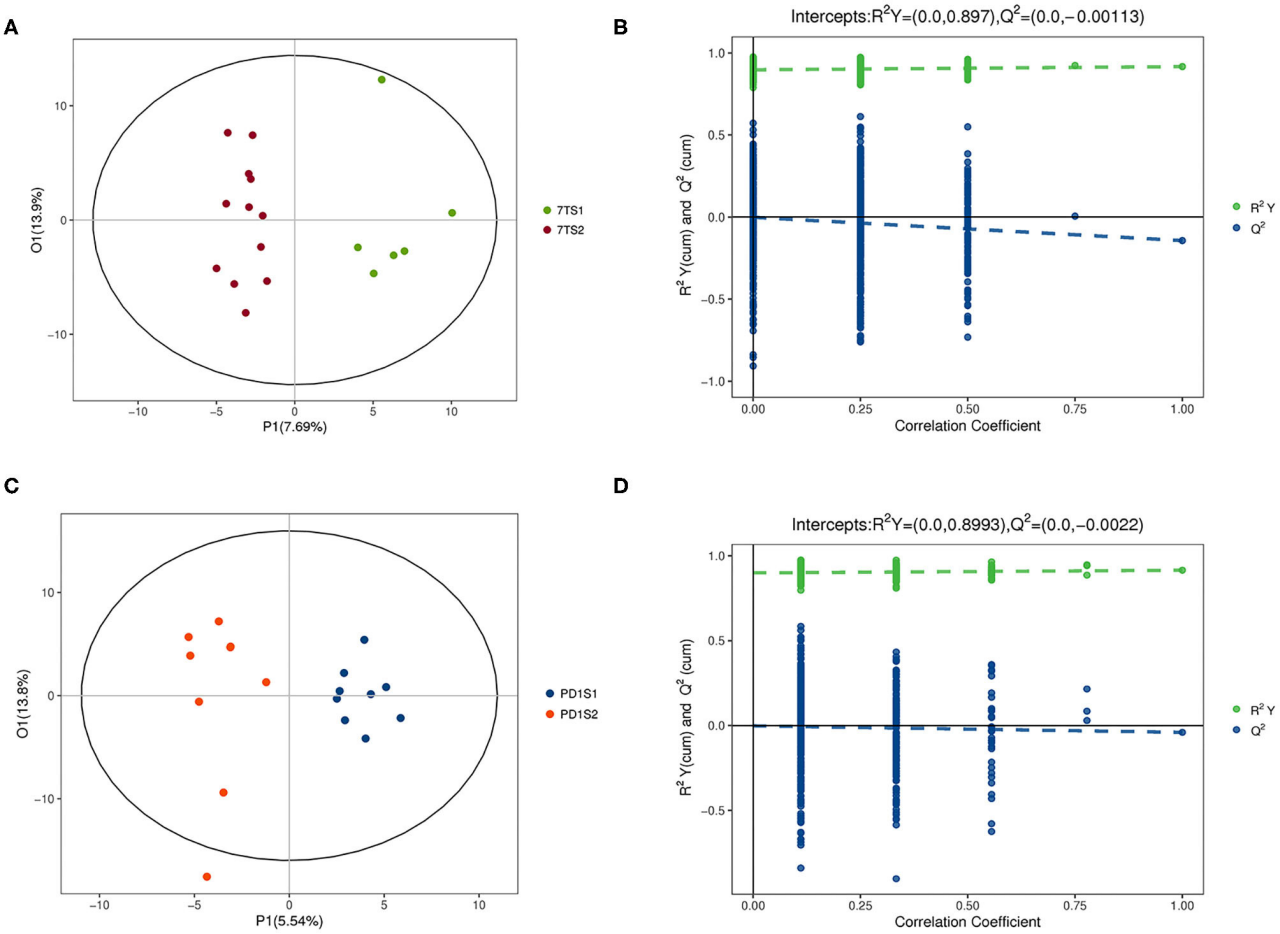
The multidimensional results in positive ionization mode are shown in this figure. **(A,B)** Are from the 7-day prognosis group; **(C,D)** are from the PD-1 expression group. **(A)** OPLS-DA score plot: the 7TS1 group vs. the 7TS2 group. **(B)** OPLS-DA validation plot intercepts: the 7TS1 group vs. the 7TS2 group; R2Y = (0.0, 0.897), Q2 = (0.0, −0.00113). **(C)** OPLS-DA score plot: the PDS1 group vs. the PDS2 group. **(D)** OPLS-DA validation plot intercepts: the PDS1 group vs. the PDS2 group; R2Y = (0.0, 0.8993), Q2 = (0.0, −0.0022).

**Figure 3:**
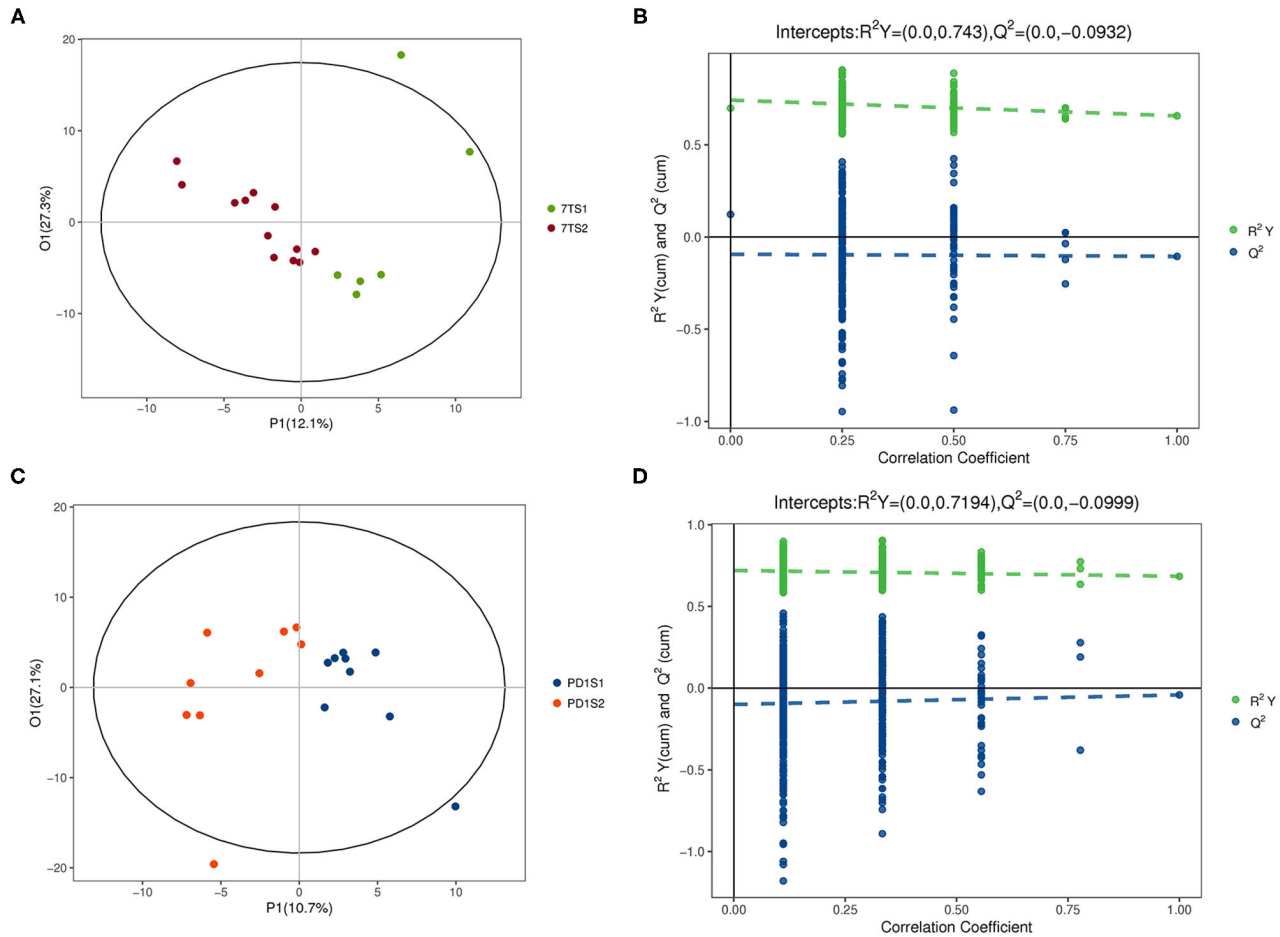
The multidimensional results in negative ionization modes are shown in this figure. **(A,B)** Are from the 7-day prognosis group; **(C,D)** are from the PD-1 expression group. **(A)** OPLS-DA score plot: the 7TS1 group vs. the 7TS2 group. **(B)** OPLS-DA validation plot intercepts: the 7TS1 group vs. the 7TS2 group; R2Y = (0.0, 0.743), Q2 = (0.0, −0.0932). **(C)** OPLS-DA score plot: the PDS1 group vs. the PDS2 group. **(D)** OPLS-DA validation plot intercepts: the PDS1 group vs. the PDS2 group; R2Y = (0.0, 0.7194), Q2 = (0.0, −0.0999).

The multidimensional results in the positive and negative ionization modes are shown in Figures 2, 3, respectively. The OPLS-DA score chart and verification chart used to perform the 7-day prognostic grouping in positive ion mode are shown in Figures 2A,B, respectively, and the results of the PD-1 expression grouping are shown in Figures 2C,D. Similarly, the OPLS-DA score chart, and verification chart used for 7-day prognostic grouping in negative ion mode are shown in Figures 3A,B, and the results for PD-1 expression grouping are shown in Figures 3C,D. The OPLS-DA score chart was used to find different metabolites through the obtained VIP values. At the same time, the validation chart verified the validity of the model.

### Combining Multidimensional and Monodimensional Analysis Methods to Identify the Respective Differential Metabolites of the 7-Day Prognosis and PD-1 Expression Groups

We obtained data sets with 324 and 257 metabolic characteristics in positive and negative ionization modes, respectively. We used a combination of multidimensional and monodimensional statistical analysis methods to obtain 16 and 8 differential metabolites between the 7-day death group and the survival group in the two modes; 5 and 8 differential metabolites were obtained between the high PD-1 expression and low PD-1 expression groups, respectively. The differential metabolites obtained for the prognosis and expression groups are shown in Tables 2, 3, respectively.

**Table 2:** In two ionization modes, one-dimensional, and multi-dimensional analysis results of differential metabolites from the 7-day prognosis group are shown.

**Ionization mode**	**Metabolites**	**7TS1 vs. 7TS2**	
		***p*-value**	**VIP**
POS	1-Pyrroline-4-hydroxy-2-carboxyl	0.042	1.968
	Alpha-dimorphecolic acid	0.028	1.526
	DG (15:0/18:3 (6Z, 9Z, 12Z)/0:0)	0.009	1.863
	DG (15:0/18:4 (6Z, 9Z, 12Z, 15Z)/0:0)	0.022	1.749
	Linezolid	0.035	1.709
	L-Targinine	0.027	2.048
	Methylcysteine	0.014	1.607
	N2-gamma-glutamylglutamine	0.026	1.708
	PC (22:1 (13Z)/20:3 (8Z, 11Z, 14Z))	0.049	1.779
	PC (24:1 (15Z)/14:1 (9Z))	0.033	2.112
	PC (P-18:0/14:0)	0.024	2.423
	PC (P-18:1 (11Z)/22:4 (7Z, 10Z, 13Z, 16Z))	0.005	2.27
	PC (P-18:1 (9Z)/16:0)	0.016	2.329
	PC (P-18:1 (9Z)/18:1 (9Z))	0.022	2.135
	L-Cyclo(alanylglycyl)	0.036	1.849
	PC (22:4 (7Z, 10Z, 13Z, 16Z)/14:0)	0.045	1.942
NEG	2-Ethyl-2-Hydroxybutyric acid	0.023	1.536
	3-Hydroxycapric acid	0.015	1.211
	D-glutamine	0.038	2.026
	Glyceraldehyde	0.047	1.339
	L-glutamine	0.022	1.784
	Maslinic acid	0.017	2.06
	Pyroglutamic acid	0.046	1.985
	Isocaproic acid	0.023	1.934

*POS, positive ionization mode; NEG, negative ionization mode; p-value, t-test p-value; VIP, VIP value of the OPLS-DA model*.

**Table 3:** In two ionization modes, one-dimensional, and multi-dimensional analysis results of differential metabolites from PD-1 expression level group are shown.

**Ionization mode**	**Metabolites**	**PD1S1 vs. PD1S2**	
		***p*-value**	**VIP**
POS	DG (18:2 (9Z, 12Z)/18:2 (9Z, 12Z)/0:0)	0.036	2.067
	DG (22:5 (4Z, 7Z, 10Z, 13Z, 16Z)/14:0/0:0)	0.023	2.353
	Isolinderanolide	0.017	2.335
	Lactosylceramide (d18:1/16:0)	0.043	2.332
	PC (P-18:0/14:0)	0.05	1.933
NEG	2-ethyl-2-hydroxybutyric acid	0.044	1.449
	5-methoxysalicylic acid	0.024	1.769
	Dehydroepiandrosterone sulfate	0.019	1.966
	Glyceraldehyde	0.038	2.302
	Indolelactic acid	0.039	1.613
	N-acetyl-a-neuraminic acid	0.031	1.425
	Paliperidone	0.02	1.635
	Ribothymidine	0.033	1.63

*POS, positive ionization mode; NEG, negative ionization mode; p-value, t-test p-value; VIP, VIP value of the OPLS-DA model*.

### Screening of Common Differential Metabolites Between the 7-Day Prognosis Group and the PD-1 Expression Group

**Figure 4:**
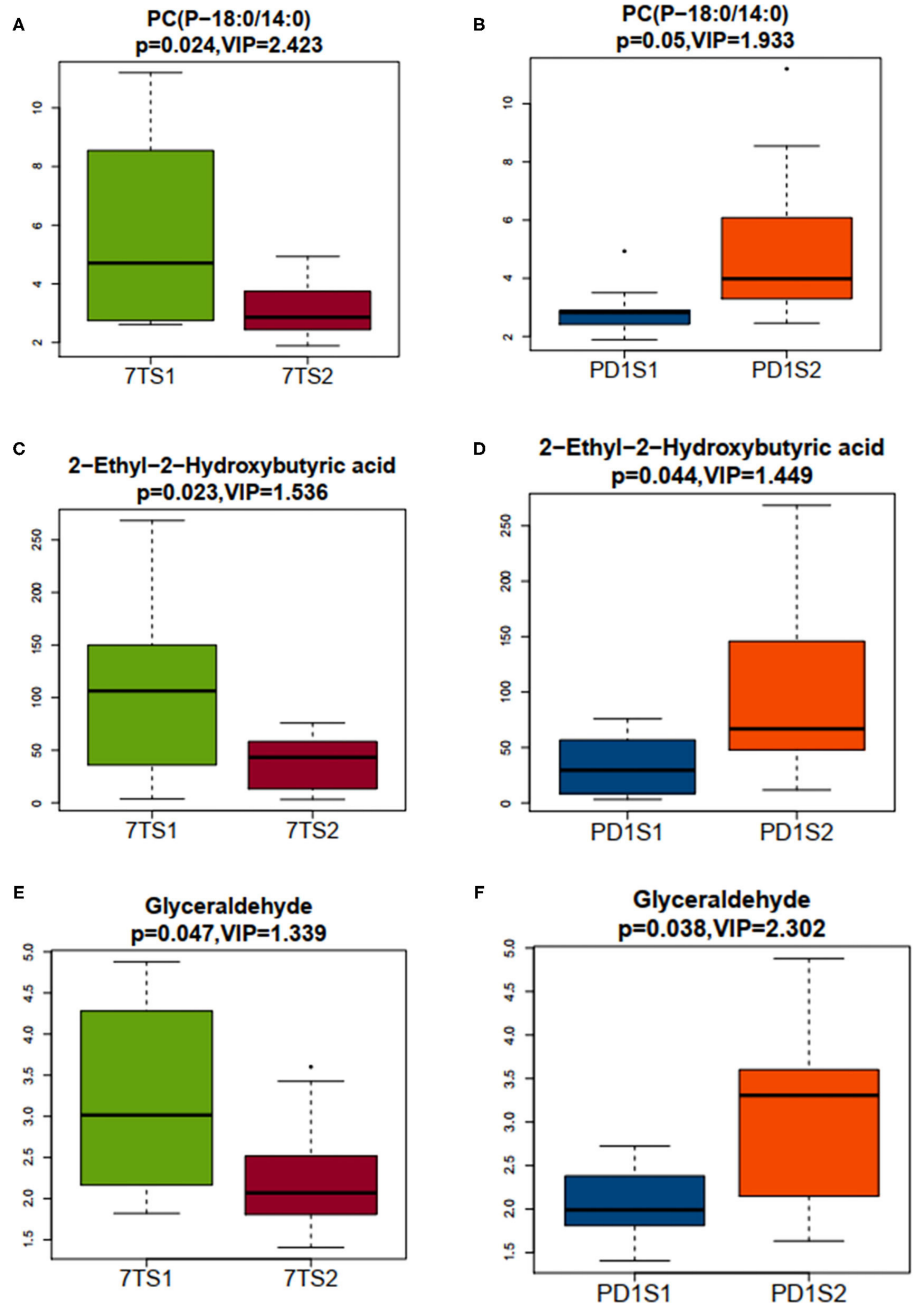
The abscissa represents the sample grouping, 7TS1 and 7TS2 represent the 7-day death group and survival group, respectively, PDS1 and PDS2 represent the high PD-1 and low PD-1 expression groups, respectively, the ordinate represents the expression of the screened differential metabolites, the box plots shows 5 statistical values (minimum value, first quartile, median, third quartile, and maximum value, that is, 5 lines from bottom to top), and VIP > 1 and *P* < 0.05 are considered significant. **(A)** The minimum value, first quartile, median, third quartile, and maximum value of PC(P-18:0/14:0) from the 7TS1 and 7TS2 groups are 2.616, 2.895, 4.708, 7.931, and 11.199 and 1.890, 2.449, 2.862, 3.624, and 4.932, respectively. **(B)** The minimum value, first quartile, median, third quartile, and maximum value of PC(P-18:0/14:0) from the PDS1 and PDS2 groups are 1.890, 2.418, 2.820, 2.897, and 3.504 and 2.459, 3.295, 3.984, 6.082, and 8.547, respectively. **(C)** The minimum value, first quartile, median, third quartile, and maximum value of 2–ethyl−2–hydroxybutyric acid from the 7TS1 and 7TS2 groups are 3.627, 43.651, 106.343, 148.977, and 268.395 and 3.182, 14.009, 43.250, 57.548, and 75.833, respectively. **(D)** The minimum value, first quartile, median, third quartile, and maximum value of 2–ethyl−2–hydroxybutyric acid from the PDS1 and PDS2 groups are 3.182, 8.516, 29.533, 56.796, and 75.833 and 11.724, 47.909, 66.808, 145.877, and 268.395, respectively. **(E)** The minimum value, first quartile, median, third quartile, and maximum value of glyceraldehyde from the 7TS1 and 7TS2 groups were 1.822, 2.3044, 3.015, 4.037, and 4.879 and 1.406, 1.809, 2.070, 2.450, and 3.426, respectively. **(F)** The minimum value, first quartile, median, third quartile, and maximum value of glyceraldehyde from the PDS1 and PDS2 groups were 1.406, 1.811, 1.991, 2.381, and 2.724 and 1.634, 2.148, 3.307, 3.602, and 4.879, respectively.

We used OPLS-DA to calculate the VIP value, which was analyzed with VIP > 1 and a *t*-test (*P* < 0.05), to screen the differential metabolites between groups. We filtered out three common differential metabolites from the two groups, namely, PC (P-18:0/14:0), 2-ethyl-2-hydroxybutyric acid, and glyceraldehyde. To visually show the expression of different metabolites in different groups, box plots of the three common differential metabolites identified are shown in Figure 4. The box plots for PC (P-18:0/14:0) in the 7-day prognosis and PD-1 expression groups are shown in Figures 4A,B, respectively; similarly, the box plots for 2-ethyl-2-hydroxybutyric acid are shown in Figures 4C,D, and the box plots for glyceraldehyde are shown in Figures 4E,F. Our results showed that PC (P-18:0/14:0) in the 7TS1 group (7-day death group) was significantly higher than that in the 7TS2 group (7-day survival group), and PC (P-18:0/14:0) in the PD1S1 group (high PD-1 expression group) was lower than PD1S2 group (low PD-1 expression group). 2-ethyl-2-hydroxybutyric acid content in the 7TS1 group was significantly higher than that in the 7TS2 group, and that in the PD1S1 group was lower than that in the PD1S2 group. At the same time, glyceraldehyde in the 7TS1 group was also higher than that in the 7TS2 group, and glyceraldehyde in the PD1S1 group was lower than that in the PD1S2 group.

### The Common Differential Metabolic Pathways Between the 7-Day Prognosis Group and PD-1 Expression Group

In this study, we performed KEGG, HMDB, PubChem, ChEBI, and METLIN pathway analysis of the differential metabolites of the two groups and found no common differential metabolic pathways for the three common differential metabolites.

### Spearman Correlation Analyses Between the Three Common Metabolites and Environmental Factors in the Two Groups

The occurrence and development of sepsis are processes related to an imbalance in proinflammatory and anti-inflammatory reactions. Inflammatory factors play an important role in the entire process, affecting the prognosis of sepsis. The expression of PD-1 is also regulated by the immune state. It is not unreasonable to speculate that there may be some correlations between inflammatory factors and PD-1. We performed Spearman correlation analyses between the different metabolites and environmental factors of the two groups. The environmental factors included IFN-γ, IL-1β, IL-2, IL-6, TNF-α, IL-10, Lac, and PCT. In positive ion mode, Spearman correlation analysis heat maps between differential metabolites and environmental factors for the 7-day prognostic group or PD-1 expression groups are shown in Supplementary Figures 2a,b, and the heat maps for Spearman correlation analyses in negative ion mode are shown in Supplementary Figures 3a,b. We focused on Spearman correlation analyses between the three common metabolites and environmental factors in the two groups. Among the metabolites, 2-ethyl-2-hydroxybutyric acid in both groups was closely related to IL-2 and Lac in negative ion mode. The content of 2-ethyl-2-hydroxybutyric acid was significantly positively correlated with the content of IL-2 and positively correlated with the content of Lac, with *P*-values of *P* < 0.01 and *P* < 0.05, respectively. Other analysis results were non-significant.

## Discussion

Metabolomics is a good analytical method to explore the pathogenesis of diseases and biomarkers. We used untargeted metabolomic methods to analyze metabolites from the six metabolite categories (acyl carnitine, amino acids, biogenic amines, glycerophospholipids, sphingolipids, and carbohydrates). Finding the common differential substances between the two groups and exploring the correlations of these substances with 7-day prognosis and PD-1 expression in sepsis were innovative.

We conducted a preliminary screening of the differences between the two groups. Most of the differential substances obtained in the 7-day prognosis group were phospholipids and amino acids, and a few were intermediate products or precursors of sugar and phospholipid metabolism. Liu et al. (19) used a serum metabolomic method and revealed that glutamine was one of the important differential metabolites between survivors and nonsurvivors of septic shock during H0-H24 and that the expression of glutamine in the death group was higher than that in the survival group. This result was consistent with the analysis results for our 7-day prognosis group. Patients with sepsis have severe metabolic changes, including alterations in the metabolism of glutamine. This molecule is involved in the regulation of nutrition and immunity in patients with sepsis and is also an important substance involved in the amino acid metabolism pathway (20–23). Therefore, glutamine is likely to be one of the promising metabolites that distinguishes prognosis at 7 days. Lactosylceramide (d18:1/16:0) (LacCer) is another differential metabolite identified in the 7-day prognosis group. LacCer is a common glycosphingolipid-derived precursor that exists throughout the body, and it is the central substance in sphingolipid synthesis and metabolism. Expressed in the cell membrane, including phagocytes, LacCer can recruit neutrophils, macrophages, and lymphocytes. It is the main signaling molecule that regulates physiological functions and various pathological processes. It has been shown to be related to inflammation and inflammation-related diseases (24–27). LacCer, as a signaling molecule, plays important roles in inflammation-induced proliferation and angiogenesis; it also plays a corresponding role in neuroinflammatory diseases. Additionally, LacCer is known to be an inflammatory factor that stimulates proinflammatory cytokine production in eye diseases. Proinflammatory cytokines can also induce the formation of ceramide, which ultimately leads to death in retinal cells (23). Daniluk et al. (15) found that the content of LacCer in children with Crohn's disease (CD) or ulcerative colitis (UC) was higher than that of children in a control group by an untargeted metabolomics method. In addition, these results indicated that LacCer might be related to inflammation and immunity. Therefore, we speculate that LacCer is likely to be another distinguishing marker for the 7-day prognosis of sepsis. Glutamine can be used as a marker to distinguish between sepsis and non-sepsis or between shock and non-shock in patients with sepsis. There are no research reports on the other differential metabolites obtained in this experiment. Therefore, future research is needed to verify these findings.

The most important finding was the identification of three common differences between the 7-day prognosis group and the PD-1 expression group, namely, PC (P-18:0/14:0), 2-ethyl-2-hydroxybutyric acid (EHBA) and glyceraldehyde (GLA). The existence of commonalities was first proposed in our research. Studies have shown that in chicken muscle metabolomic analysis, the most significantly different metabolites are phospholipids, including phosphatidylcholine (PC) and phosphatidylethanolamine (PE) (28). PC (P-18:0/14:0) can be used as a metabolic marker for the response of *Streptococcus salivarius* to a sudden drop in salinity (29). EHBA can be used as a precursor in the synthesis of halogenated lipids and amino acids. EHBA is usually used to assist in the extraction of metals such as neodymium, terbium, and thulium by the P 204 (HDEHP) liquid method. In existing research, the above two substances have not been found to be markers of sepsis or other diseases. GLA is the simplest aldose and is produced by glucose degradation and glycosylation, and some studies have noted that GLA can distinguish diabetic patients from healthy people (30). Glyceraldehyde-3-phosphate dehydrogenase (GAPDH) is an enzyme related to energy metabolism in the body and is one of the key enzymes involved in the glycolytic pathway. When the activity of GAPDH decreases, GLA accumulation can increase. GAPDH acts on immune cells and regulates their immune and inflammatory responses (31). GLA has not previously been proposed to be a marker of sepsis or other diseases in existing research.

Another important result is that the levels of IL-2 and Lac were positively correlated with the level of EHBA by Spearman correlation analysis. IL-2 is mainly produced by activated T cells, which can promote the growth, proliferation, and differentiation of lymphocytes. It plays an important role in the body's immune response and in antiviral infection. It can stimulate the activation of antigen-specific cells and mitogenic factors. T cell proliferation can activate T cells and promote cytokine production. After PD-1 binds to its ligand, the subsequent signaling inhibits the proliferation of T cells; the production of cytokines, such as IL-2 and IFN-γ; the proliferation and differentiation of B cells; and the secretion of immunoglobulins. These events play negative roles in regulating the immune response (32). Lac is the final product of anaerobic glucose metabolism. The blood Lac level has traditionally been interpreted as a marker of tissue hypoxia. Patients with sepsis, especially those with septic shock, usually have elevated Lac levels in the body. Lac is often used clinically to assess the severity and prognosis of sepsis/septic shock. The activation of immune cells requires aerobic glycolysis. Lac produced by anaerobic glycolysis may cause immunosuppression during sepsis. Lac can be used as a new biomarker related to the downregulation of the immune system in patients with sepsis (33, 34). In this study, the 7-day death group and the low PD-1 expression group exhibited higher EHBA levels than the 7-day survival group and the high PD-1 expression group, respectively. EHBA levels were higher in the 7-day death group and the low PD-1 expression group, and the IL-2 and Lac levels were also higher. We speculate that EHBA may be affected by the environmental factors IL-2 and Lac to affect PD-1 expression on the surface of T cells, which in turn affects the 7-day prognosis of sepsis patients. The results of correlation analyses between the other two common differential metabolites and environmental factors were negative, which may be due to the small sample size and individual differences. These findings require further research and verification.

The limitation of this study was the small number of participants. Therefore, we propose to verify the differential substances found in our experiments in future multicenter clinical studies.

In conclusion, the metabolites of patients with sepsis change substantially, with changes mainly related to the metabolic pathways of amino acid metabolism, glycolysis, and fat catabolism. The common differential metabolites in the 7-day prognosis group and PD-1 expression group were found in the plasma of patients diagnosed with sepsis within 24 h. These common metabolites may affect PD-1 expression and 7-day prognosis. They may also be related factors that affect the 7-day prognosis by regulating PD-1 expression in patients with sepsis and may be used as new biomarkers for sepsis diagnostic and prognostic assessments. IL-2 and Lac, which were identified by Spearman correlation analysis, may play important roles in the expression of PD-1 on the surface of T cells and affect the prognosis of patients with sepsis.

## Data Availability Statement

The original contributions presented in the study are included in the article/Supplementary Material, further inquiries can be directed to the corresponding author/s.

## Ethics Statement

The studies involving human participants were reviewed and approved by Medical Ethics Committee of the Harbin Medical University Cancer Hospital (No.KY2018-02). The patients/participants provided their written informed consent to participate in this study.

## Author Contributions

YB and HW carried out the study and wrote the main manuscript. XM and CH helped carry out the study and drafted the chart. XJ, JZ, and YL carried out the study and data analysis. YP and MY helped to draft the charts and figures. KY and CW conceived the study, participated in its design and coordination, and helped draft the manuscript. All authors read and approved the final manuscript.

## Conflict of Interest

The authors declare that the research was conducted in the absence of any commercial or financial relationships that could be construed as a potential conflict of interest.

## Supplementary Material

Supplementary Figure 1 The flow gating strategy for PD-1 expression on CD4+ T surface is shown in figure. **(a,b)** Are examples of 7TS1 and 7TS2 group, respectively. FSC is forward scatter and SSC is side scatter. FITC is labeled CD3+ T cells, PerCP is labeled CD4+ T cells, V500-C is labeled CD8+ T cells, and PE is labeled PD-1.

Supplementary Figure 2 Spearman correlation analysis heat map between environmental factors and differential metabolites obtained in positive ion mode. The abscissa represents the environmental factor, and the ordinate represents the metabolite. The depth of the color visually shows the correlation between the metabolite and the environmental factor; at the same time, a test of the significance of the correlation was performed. When *P* < 0.05, the significance is marked with +; when *P* < 0.01, ^*^indicates significance. **(a)** Spearman correlation analysis heat map between environmental factors and differential metabolites in the 7-day prognostic group. **(b)** Spearman correlation analysis heat map between environmental factors and differential metabolites in the PD-1 expression group.

Supplementary Figure 3 Spearman correlation analysis heat map between environmental factors and differential metabolites obtained in negative ion mode. The abscissa represents the environmental factor, and the ordinate represents the metabolite. The depth of the color visually shows the correlation between the metabolite and the environmental factor; at the same time, a test for significance of the correlation was performed. When *P* < 0.05, the significance is marked with +; when *P* < 0.01, ^*^indicates significance. **(a)** Spearman correlation analysis heat map between environmental factors and differential metabolites in the 7-day prognostic group. **(b)** Spearman correlation analysis heat map between environmental factors and differential metabolites in the PD-1 expression group.
