# Supplementary figures and images for: Untargeted Metabolomic Profiling of the Correlation Between Prognosis Differences and PD-1 Expression in Sepsis: A Preliminary Study
Source: Front Immunol. 2021 Apr 1;12:594270. doi: 10.3389/fimmu.2021.594270 (PMC8046931; doi:10.3389/fimmu.2021.594270)

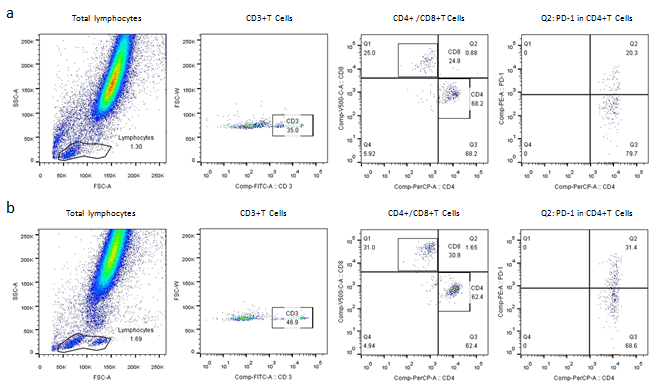

Supplement: Supplementary Figure 1 — The flow gating strategy for PD-1 expression on CD4+ T surface is shown in figure. (a,b) Are examples of 7TS1 and 7TS2 group, respectively. FSC is forward scatter and SSC is side scatter. FITC is labeled CD3+ T cells, PerCP is labeled CD4+ T cells, V500-C is labeled CD8+ T cells, and PE is labeled PD-1. [file Image_1.TIF]

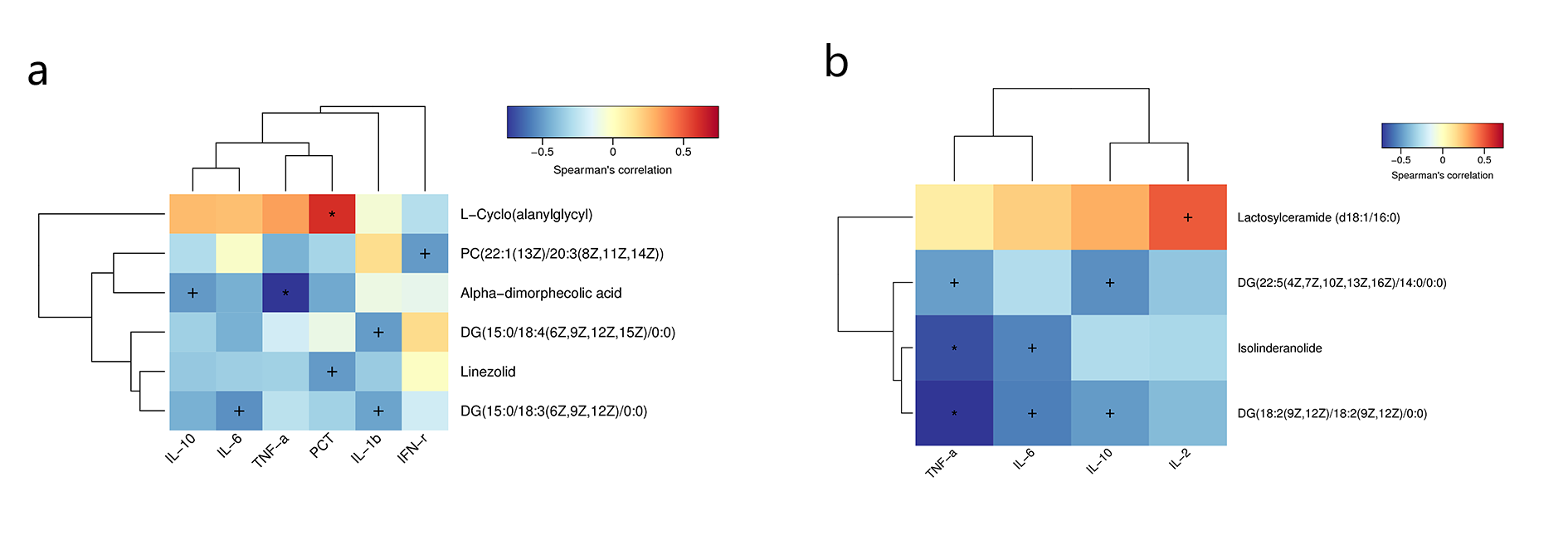

Supplement: Supplementary Figure 2 — Spearman correlation analysis heat map between environmental factors and differential metabolites obtained in positive ion mode. The abscissa represents the environmental factor, and the ordinate represents the metabolite. The depth of the color visually shows the correlation between the metabolite and the environmental factor; at the same time, a test of the significance of the correlation was performed. When P < 0.05, the significance is marked with +; when P < 0.01, *indicates significance. (a) Spearman correlation analysis heat map between environmental factors and differential metabolites in the 7-day prognostic group. (b) Spearman correlation analysis heat map between environmental factors and differential metabolites in the PD-1 expression group. [file Image_2.TIF]

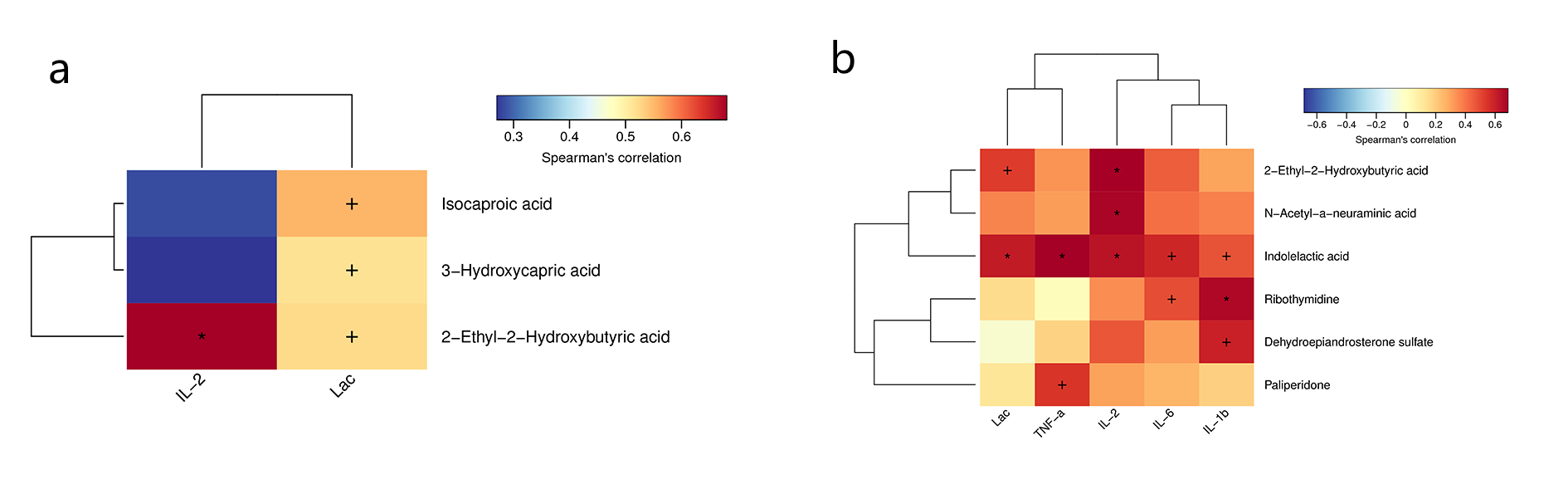

Supplement: Supplementary Figure 3 — Spearman correlation analysis heat map between environmental factors and differential metabolites obtained in negative ion mode. The abscissa represents the environmental factor, and the ordinate represents the metabolite. The depth of the color visually shows the correlation between the metabolite and the environmental factor; at the same time, a test for significance of the correlation was performed. When P < 0.05, the significance is marked with +; when P < 0.01, *indicates significance. (a) Spearman correlation analysis heat map between environmental factors and differential metabolites in the 7-day prognostic group. (b) Spearman correlation analysis heat map between environmental factors and differential metabolites in the PD-1 expression group. [file Image_3.TIF]
